# Long term outcomes of colon polyps with high grade dysplasia following endoscopic resection

**DOI:** 10.1186/s12876-020-01499-2

**Published:** 2020-11-10

**Authors:** Jia-Jang Chang, Cheng-Hung Chien, Shuo-Wei Chen, Li-Wei Chen, Ching-Jung Liu, Cho-Li Yen

**Affiliations:** 1 Division of Hepatogastroenterology, Keelung Chang Gung Memorial Hospital, No. 222, Mai Chin Road, Keelung, 204 Taiwan; 2 Keelung Division, Chang Gung Memorial Hospital, Keelung, Taiwan

**Keywords:** Colonoscopy, Colon polyp, High grade dysplasia, Surveillance

## Abstract

**Background:**

The risk of recurrent colonic adenoma associated with high-grade dysplasia (HGD) colon polyps at baseline colonoscopy remains unclear. We conducted a clinical cohort study with patients who underwent polypectomy during screen colonoscopy to assess recurrent colonic adenoma risk factors.

**Methods:**

11,565 patients at our facility underwent screen colonoscopy between September 1998 and August 2007. Data from patients with HGD colon polyps who had undergone follow-up colonoscopy were included for analysis.

**Results:**

Data from 211 patients was included. Rates of metachronous adenoma and advanced adenoma at follow-up were 58% and 20%, respectively. Mean follow-up period was 5.5 ± 1.8 (3–12) years. Univariate logistic regression analysis revealed that an adenoma count of ≥ 3 at baseline colonoscopy was strongly associated with overall recurrence, multiple recurrence, advanced recurrence, proximal recurrence, and distal adenoma recurrence with odds ratios of 4.32 (2.06–9.04 95% CI), 3.47 (1.67–7.22 95% CI), 2.55 (1.11–5.89 95% CI), 2.46 (1.16–5.22 95% CI), 2.89 (1.44–5.78 95% CI), respectively. Multivariate analysis revealed gender (male) [*P* = 0.010; OR 3.09(1.32–7.25 95% CI)] and adenoma count ≥ 3 [*P* = 0.002; OR 3.08(1.52–6.24 95% CI)] at index colonoscopy to be significantly associated with recurrence of advanced adenoma.

**Conclusion:**

Recurrence of colonic adenoma at time of follow-up colonoscopy is common in patients who undergo polypectomy for HGD colon adenomas during baseline colonoscopy. Risk of further developing advanced adenomas is associated with gender and the number of colon adenomas present.

## Background

Colorectal cancer (CRC) is the third leading cause of cancer-related death in Taiwan [1]. CRCs originate from the initially benign colon adenomas that subsequently undergo an adenoma-carcinoma transition sequence. Polypectomy interrupts this sequence and reduces the incidence of CRC [2–5]. The occurrence of CRC can also be effectively prevented by detecting and excising advanced adenomas, which are defined as larger than 10 mm, displaying a high grade dysplasia (HGD), and/or ≥ 20% villous [6]. The 5-year rate of recurrence for colonic adenoma following polypectomy range between 29–58% [5, 7], and previous studies have suggested that adenoma traits at index colonoscopy are closely related to recurrence. Specifically, location, size, histological type, presence of atypia, and number of adenomas detected at index colonoscopy are known risk factors for adenoma recurrence [8–11].

Whether advanced adenoma (villous adenoma, severe dysplasia, and/or size ≥ 10 mm) at index colonoscopy exhibit more aggressive behavior and earlier recurrence than typical adenoma is still unclear [11]. A univariate analysis using NCI Pooling Project data, adenomas with HGD were shown to be strongly associated with risk of advanced neoplasia by follow-up colonoscopy (OR, 1.77; 95% CI, 1.41–2.22) [11]. However, this finding was not duplicated in other studies. Indeed, only a few studies have yet to address the long- term outcome of patients with HGD colon polyps following polypectomy [12, 13]. We therefore conducted this retrospective cohort study with the primary goals of elucidating a general recurrence rate for advanced neoplasia in patients with HGD polyps at baseline screening, as well as the relationship between baseline endoscopic findings and risk of developing advanced neoplasia.

## Methods

### Data selection

After obtaining approval from the Institutional Review Board of Keelung Chang Gung Memorial Hospital (IRB No. 104-6993D), an extensive review of patient records was undertaken to gather relevant colonoscopy and pathology reports. We gathered a total of 11,565 relevant cases that underwent colonoscopy between September of 1998 and August of 2007. The procedures were performed by experienced endoscopists using the Olympus EVIS CF260 AI or EVIS CF 260 AL variable stiffness scope.

Patients with adenoma at screening and who underwent subsequent surveillance colonoscopy were filtered, and cases involving HGD colon adenoma were selected for review.

Cases with a history of typical or metachronous colon cancer at time of index study, inflammatory bowel disease (including Crohn’s disease or ulcerative colitis), familial adenomatous polyposis (FAP), a lack of surveillance total colonoscopy within three years of screen polypectomy, incomplete colonoscopy results, or other disease that led to death within the study period were excluded.

A complete colon visualization was required for patients who underwent colonoscopy at our facility. Patient who had colonic neoplastic lesions at colonoscopy underwent routine polypectomy using biopsy forceps (small lesions less than 5 mm), cold snare polypectomy, hot snare polypectomy, or endoscopic submucosal resection. The gross appearance, size, histology, and location of the lesions at baseline colonoscopy were compared between recurrence and recurrence-free groups to elucidate possible risk factors of future adenoma recurrence. Adenomas were categorized as protruding (0-Ip), subpedunculated (0-Ips), sessile (0-Is), lateral spreading (0-Iia), or depressed (0-IIc) according to the Paris endoscopic classification of superficial neoplastic lesions [13]. The size, morphology, and location of the adenomas were also recorded. Adenoma size was measured using open biopsy forceps (diameter = 8 mm) or by ruler for resected specimen. The lesion site was defined as either proximal colon (caecum, ascending colon, hepatic flexure and transverse colon) or distal colon (splenic flexure, descending colon, sigmoid colon and rectum).

Excised adenomas underwent pathological examination. Advanced adenomas were defined by size ≥ 10 mm, presence of villous or villotubular histology (≥ 20% villous component), and/or possessing a grade of dysplasia (severe dysplasia or intramucosal carcinoma). Polyps were defined as tubular, tubulovillous, villous adenoma, or serrated according to histological analysis.

Follow-up colonoscopy was performed 6 to 12 months after index colonoscopy if adenomas were removed via the piecemeal method or if incomplete resection was suspected. Patients who underwent surveillance colonoscopy within three years of initial polypectomy were eligible for inclusion. Clinical traits including gender, age, indication of colonoscopy, family history of colon cancer, tumor number, size, site, and histology were considered.

Continuous variables were expressed as mean ± standard deviation (mean ± SD) and categorical variables as frequencies or percentages. The independent t-test and chi-square test were used to compare demographic categories among all cases with high grade dysplasia (Table 1) and to compare recurrence vs, recurrence-free groups (Table 2). Univariate logistic regression analysis was performed to obtain the odds ratios of all predictors for polyp recurrence (Tables 3 and 4). Kaplan–Meier method and log rank test were used to compare the recurrence rates of adenomas based on the initial polyp counts (Fig. 2). Multivariate logistic regression analysis was performed to determine the risk of adenoma and advanced colorectal neoplasia recurrences from baseline endpoints (Table 5). A *p* value < 0.05 was considered statistically significant and all calculations were two sided. All statistical analyses were performed using IBM SPSS 22.0 (IBM Inc., Armonk, New York).

**Table 1:** Baseline characteristics of HGD in adenomas and recurrence rates

Characteristics	Number (%)	Adenoma recurrence (%)
Gender	M: 129 (61.1) F: 82 (38.9)	81 (62.8) 42 (51.2)
Family history of CRC^§^	11 (5.2)	4 (36.4)
Stool OB positive^§§^	99 (46.9)	44 (44.4)
Pedunculated(0-Ip)*	72 (34.1)	37 (51.4)
Subpedunculated(0-Ips)* Sessile(0-Is)* Lateral spreading(0-IIa)* Depressed (0-IIc)*	97 (46.0) 23 (10.9) 18 (8.5) 1 (0.5)	56 (57.7) 18 (78.2) 11 (61.1) 1 (100)
Tubular adenoma Tubulovillous/Villous adenoma Serrated adenoma	154 (73.0) 53 (25.1) 4 (1.9)	94 (61.0) 26 (49.1) 3 (75)
Distal colon** Proximal colon*** Both distal and proximal	119 (56.4) 28 (13.3) 64 (30.3)	65 (54.6) 14 (50.0) 44 (68.8)
1 Polyp	93 (44.1)	40 (43.0)
2 Polyps	58 (27.5)	37 (63.8)
≥3 Polyps	60 (28.4)	46 (76.7)

^§^Family history of CRC (first relatives)

^§§^Positive fecal immunochemical test

^*^The Paris endoscopic classification of superficial neoplastic lesion

**Distal colon (splenic flexure, descending colon, sigmoid colon and rectum)

***Proximal colon (caecum, ascending colon, hepatic flexure and transverse colon)

**Table 2:** Clinical characteristics of 211 patients with or without adenoma recurrence

	Recurrence free (n = 88)	Recurrence (n = 123)	*P* value
Age, years	64.06 ± 11.87	63.89 ± 11.8	0.828
Size of polyp, cm	1.91 ± 1.11	1.83 ± 1.2	0.576
Baseline polyp No	1.68 ± 1.09	2.46 ± 1.57	0.03
Male gender	48 (54.5%)	81 (63.9%)	0.097
Morphology			0.201
Ip*	35	37	
0-Ips**	41	56	
0-Is***	5	18	
Lateral spreading (0-IIa)	7	11	
Depressed(0-IIc)	0	1	
Histology			0.284
Tubular	60	94	
Villous/villotubular	27	26	
Serrated	1	3	
Location			0.706
Distal	54	65	
Proximal	14	14	
Both	20	44	

Ip* pdunculated, 0-Ips** subpedunculated, 0-Is*** sessile

**Table 3:** The impact of number and location of polyps at baseline on the recurrence of polyps at follow up colonoscopy

Recurrence	Distal colon polyps at baseline					Proximal colon polyps at baseline					
	< 3 adenomas*(51)		≥ 3 adenoma*(14)			< 3 adenomas*(26)			≥ 3 adenomas*(32)		
	%	OR	%	OR	*P*	%	OR	*P*	%	OR	*P*
Overall	49	1	93	13.42 (1.69–106.51)	0.01	55	1.23 (0.61–2.48)	0.56	71	2.41 (1.12–5.184)	0.02
Multiple	35	1	60	4.53 (1.45–14.19)	< 0.01	32	1.41 (0.66–3.04)	0.37	42	2.24 (1.05–4.79)	0.04
Distal	36	1	87	11.02 (2.33–52.17)	< 0.01	36	0.99 (0.48–2.05)	0.98	47	1.58 (0.76–3.28)	0.22
Proximal	22	1	27	1.27 (0.36–4.45)	0.71	30	1.46 (0.67–3.33)	0.34	42	2.40 (1.11–5.20)	0.03
Advanced	12	1	40	3.71 (1.10–12.50)	0.03	23	1.88 (0.76–4.66)	0.18	29	2.28 (0.93–5.56)	< 0.01

^*^() patient number. OR odd ratio, Overall = overall adenoma recurrence, Multiple = multiple adenoma recurrence

Distal = distal adenoma recurrence, Proximal = proximal adenoma recurrence, Advanced = advanced adenoma recurrence

**Table 4:** Baseline clinical characteristics and risk for recurrent adenoma upon univariate analysis

	Overall adenomas recurrence		Advanced adenomas recurrence	
	OR (95% CI)	P value	OR (95% CI)	*P* value
Age in years	1.00 (0.98–1.03)	0.928	1.01 (0.98–1.04)	0.441
Male gender (vs female)	1.75 (0.99–3.06)	0.052	3.44 (1.51–7.87)	0.003
Size of polyp (≥ 10 mm vs < 10 mm)	0.89 (0.44–1.83)	0.758	0.95 (0.40–2.26)	0.909
Adenoma number (≥ 3 vs < 3)	3.16 (1.60–6.22)	0.001	2.45 (1.22–4.93)	0.012
FH^*^(positive vs negative)	1.27 (0.36–4.47)	0.713	0.38 (0.05–3.02)	0.358
Stool OB^**^ (Positive vs negative)	0.81 (0.47–1.40)	0.448	1.10 (0.56–2.15)	0.778
Pathology (Non-tubular vs tubular)	0.63 (0.34–1.17)	0.143	0.94 (0.44–2.02)	0.873
Location (Distal vs Proximal)	1.13 (0.60–2.15)	0.707	0.49 (0.34–1.02)	0.056

FH family History, *positive family history of CRC (first relatives), OB occult blood, **positive fecal immunochemical test

**Table 5:** Baseline characteristics and risk ratios for recurrent adenomas upon multivariate analysis

	Overall adenomas recurrence		Advanced adenomas recurrence	
	OR (95% CI)	*P*	OR (95%CI)	*P*
Age in years	0.99 (0.97–1.02)	0.661	1.00 (0.97–1.04)	0.795
Male vs female	1.59 (0.87–2.90)	0.127	3.09 (1.32–7.25)	0.010
Size ≥ 10 mm vs < 10 mm	0.76 (0.35–1.65)	0.492	0.83 (0.32–2.18)	0.704
Adenoma number ≥ 3 vs < 3	3.08 (1.52–6.24)	0.002	2.11 (1.00–4.43)	0.049
FH^*^ positive vs negative	1.24 (0.33–4.69)	0.755	0.45 (0.05–3.84)	0.592
Stool OB^**^ positive vs negative	0.90 (0.50–1.62)	0.720	1.22 (0.59–2.55)	0.611
Non-tubular vs tubular	0.59 (0.30–1.34)	0.404	1.07 (0.47–2.43)	0.871
Distal vs proximal location	1.34 (0.67–2.68)	0.746	0.52 (0.23–1.156)	0.109

FH family history, *positive family history of CRC (first relatives), OB occult blood, **positive fecal immunochemical test

## Results

Of the 11,565 patients who underwent colonoscopy, colon adenomas were found in 4149 (35.9%). Of these 4149 patients, 258 (6%) had advanced adenomas with HGD.

**Fig. 1:**
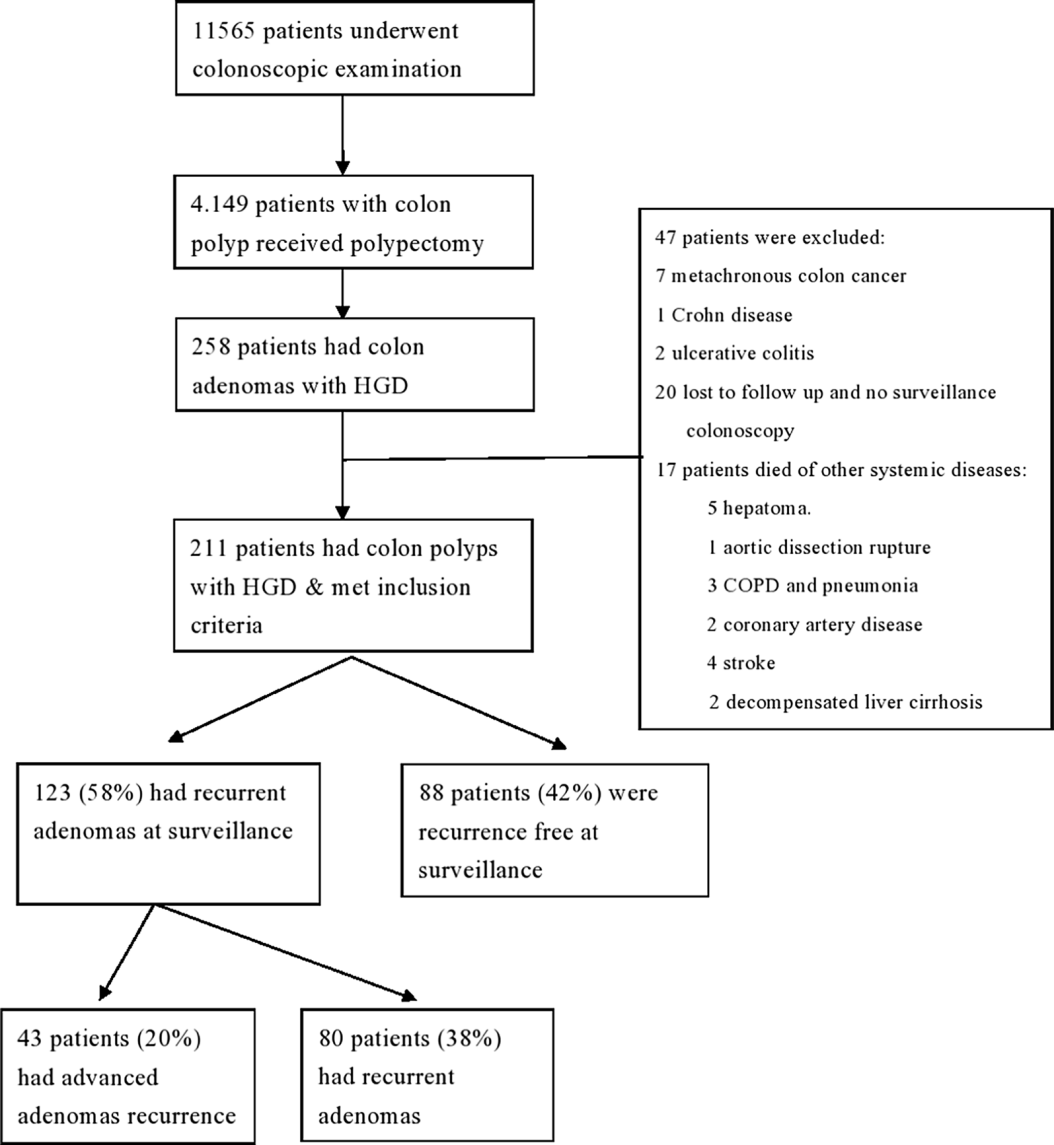
Diagram outlining selection and exclusion criteria

47 of 258 patients failed to complete surveillance colonoscopy and were excluded. Figure 1 shows selection and exclusion results. A total of 211 patients were included for analysis. Mean age was 64 ± 12 years, and mean follow-up period was 5.5 ± 1.8 (3–12) years after index colonoscopy. 129 patients were male and 82 patients were female. 123 patients (58%) experienced recurrent adenoma at surveillance colonoscopy.

**Fig. 2:**
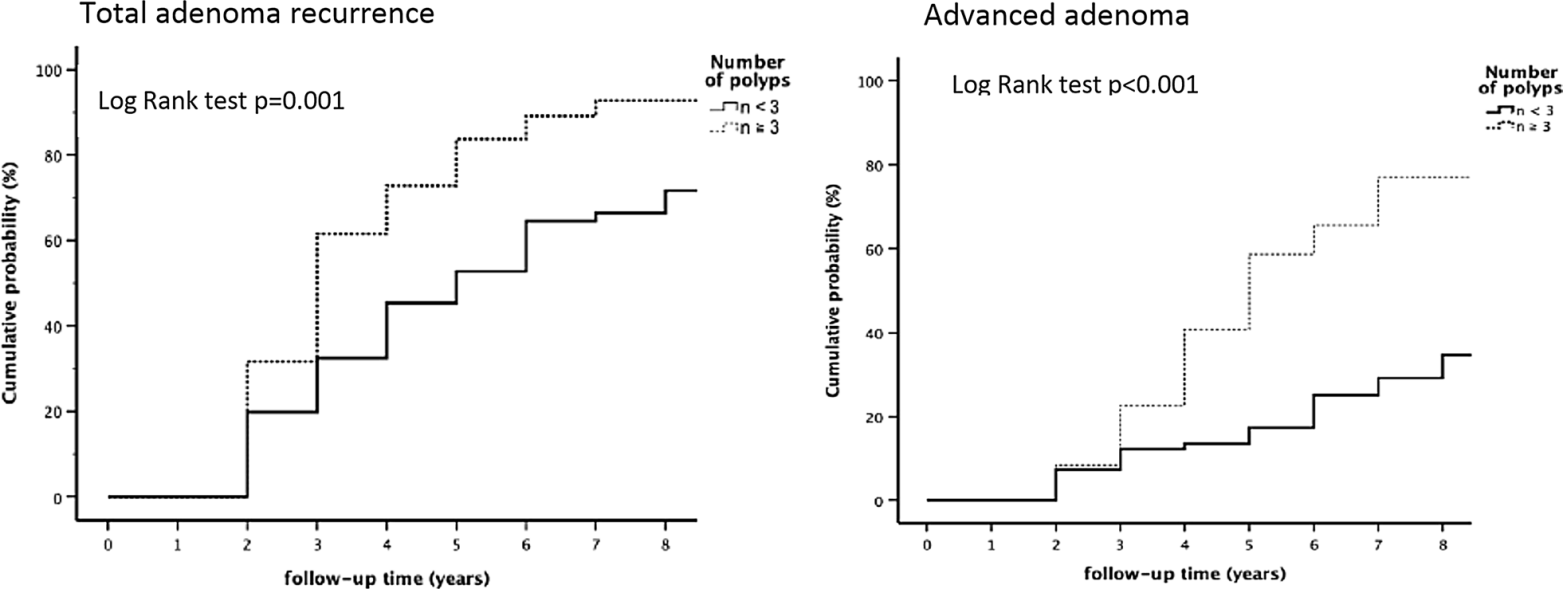
Kaplan–Meier curves presenting the cumulative rates of recurrence according to initial polyp counts. There were significant differences between the two groups in total adenoma and advanced adenoma recurrence according to the log-rank test analysis

There was no statistical difference between recurrence and recurrence-free groups in terms of gross appearance, histological finding, location, or size of adenoma at baseline colonoscopy (Table 2). The mean number of polyps 2.46 at index colonoscopy was associated with future adenoma recurrence (*P* = 0.03) (Table 2). A further analysis with Kaplan Meier method revealed a significant greater recurrence rate in patients with polyp number ≥ 3 than those with polyp number ≤ 2 (Fig. 2). The cumulative total adenoma and advanced adenoma recurrence at 3 years and 5 years were 32.5%, 52.7% and 12.2%,17.3% for polyp number < 3 in comparison to 61.6%, 83.7% and 22.7%, 58.6% for polyp number ≥ 3 (*P* < 0.001).

Subgroup analysis of polyp number and site on recurrence revealed that patients with ≥ 3 lesions experienced a greater risk of metachronous adenoma recurrence (Table 3). For distal colon polyps numbering ≥ 3 at index colonoscopy, rate of overall recurrence, multiple recurrence, distal colon recurrence, proximal colon recurrence, and advanced adenoma recurrence were 93%, 60%, 87%, 27%, and 40%, respectively. In contrast, distal polyps numbering < 3 revealed an overall recurrence rate, multiple recurrence rate, distal colon recurrence rate, proximal colon recurrence rate, and advanced adenoma recurrence rate of 49%, 35%, 36%, 22%, and 12%, respectively. For lesions located in the proximal colon at index colonoscopy, a similar odds ratio was also observed in the ≥ 3 group but not in the < 3 group (Table 3). The odds ratios for overall recurrence, multiple recurrence, distal recurrence, proximal recurrence, and advanced adenoma recurrence with number ≥ 3 and < 3 were 13.42 (1.69–106.51), 4.53 (1.45–14.19), 11.02(2.33–52.17), 1.26 (0.36–4.45), and 3.71 (1.10–12.50), respectively (Table 3).

Univariate logistic regression analysis revealed lesions > 3 in number at baseline colonoscopy was strongly associated with risk of overall recurrence, multiple recurrence, advanced recurrence, proximal recurrence, and distal adenoma recurrence (Table 4) with odds ratios of 4.32 (2.06–9.04 95% CI), 3.47 (1.67–7.22 95% CI), 2.55 (1.11–5.89 95% ), 2.46 (1.16–5.22 95% CI), 2.89 (1.44–5.78 95% CI), respectively. Size, pathological characteristics, or site of adenoma were not associated with risk of overall recurrence, multiple recurrence, proximal recurrence, distal recurrence, or advanced adenoma recurrence (Table 4). Multivariate logistic regression analysis revealed that polyp number > 3 was associated with risk of overall polyp recurrence [*P* = 0.002; OR 3.08 (1.52–6.24 95%CI)] and advanced polyp recurrence at surveillance examination [*P* = 0.049; OR 2.11 (1.00–4.43 95%CI)]. Gender (male) [*P* = 0.010; OR 3.09 (1.32–7.25 95% CI)] was also associated with advanced polyp recurrence (Table 5). Of the 211 patients who underwent surveillance colonoscopy, 43 patients (20%) developed recurrent advanced colon polyps. No patients in our study developed interval colorectal adenocarcinoma.

## Discussion

The rates of recurrent metachronous adenoma and advanced adenoma after polypectomy for colon polyps with HGD were 58% and 20%, respectively. Age, gender, stool occult blood, size of polyp, morphology, pathology, and site of polyp did not differ between patients with recurrent adenoma and those without.

The odds ratios of developing metachronous adenoma and advanced adenoma were 4.32 (2.06–9.04 95% CI) and 2.55 (1.11–5.89 95% CI), respectively, upon comparing subjects with polyps ≥ 3 and ≤ 2 in number at index colonoscopy.

No metachronous adenocarcinoma was observed during the analysis period. The incidence rate of adenoma was 35.9% among those who underwent screening colonoscopy. This rate is higher than similar patient population in Western countries, which have been cited as low as 20% [14]. This rate is also higher than Taiwanese (8.13%) and other ethnically Chinese groups (16.5%) [15–17]. It should be noted that cases included in our study were mostly department referrals on complaint of stool occult blood or gastrointestinal symptoms.

Polypectomy is 50–80% successful in avoiding colon cancer, and interval cancers are located mainly in the right colon [18–20]. Interval carcinoma occurs more frequently in the right colon due to incomplete colonoscopy, often resulting from poor endoscopic technique [21], incomplete removal of polyps [22], difficulty in visualization of smaller adenomas in the right colon [23, 24], or sessile serrated adenoma in the proximal colon.

At screening colonoscopy, the prevalence of polyps with HGD was 2.2% (258/11,565) in our data set, which is slightly lower than 3.5% in the general ethnically Chinese population [26]. There was no interval cancer seen during the follow up period in our study given that we performed the second look colonoscopy 6 to 12 months after piecemeal resection for advanced polyps or suspected incomplete resection to ensure complete resection of polyps and to decrease the rate of undetected adenomas at time of index colonoscopy. This protocol has been suggested by some endoscopists when using the piecemeal method [25, 26]. Recurrent adenomas after polypectomy are reported to occur about 36 to 64% within 2 to 6 years [12, 27–30]. In our study, the overall recurrence rate of metachronous adenomatous polyps was 58%, which is similar to that of 64% in Toll*'*s study [12]. The rate of recurrent advanced adenomas was 20% in our study, which is lower than the 40% from Toll’s report.

HGD in adenoma is in line with the adenoma-adenocarcinoma sequence, and is a precursor of adenocarcinoma [31]. However, the risk of future advanced adenomas in relation to HGD at index colonoscopy was reported to be small and variable.

In a meta-analysis study by Saini [32], patients with HGD in polyps experience a 1.84-fold risk of developing advanced adenoma (95% CI 1.06–3.19) compared to those without HGD. Some other studies, including a randomized controlled trial, revealed no association of HGD with subsequent advanced adenomas during surveillance colonoscopy [33–36].

Two meta-analyses [37, 38] have shown that the presence of HGD is slightly associated with future advanced adenoma. Upon multivariate analysis, the presence of HGD was not found to confer recurrence of metachronous adenoma. Most advanced adenomas are ≥ 1 cm in size [39]. The risk for metachronous recurrence may dependent on the number of colon polyps and gender, as shown in our study. Moreover, this rationale is also applicable to the association of villous histology in resected adenoma with the risk of recurrent advanced adenoma. Another cohort study found that the presence of villous adenoma at baseline colonoscopy carried a 1.8-fold risk of future adenoma [33]. Two other studies, including one meta-analysis and a pooled analysis, found no association between villous histology and future adenoma development [14, 32]. Such results are in line with the findings of our study. The size of colonic adenoma has been shown to be closely related to HGD and cancer-like histological features [40]. HGD and/or prominent villous components are seen in 87.5% of large polyps (≥ 1 cm) [95% CI = 86–89.4]) [41]. Moreover, the risk of recurrent advanced adenoma was found to correspond with the size of polyps at index colonoscopy. Martinez [14] found that adenoma size ranges 1.0–1.9 cm and ≥ 2.0 cm at baseline colonoscopy carried a relative risk of 2.3 and 3.0 respectively for developing recurrent advanced adenoma compared to a size of 0.5 cm. Additionally, 4 other studies found increased risk of advanced adenoma in colonic adenomas greater than 1 cm at baseline [22, 32, 34–36]. The relative risk of polyps with HGD at baseline was 6.87 (95% CI 2.61–18.07) for interval advanced neoplasia in a study by Lieberman, in which 6 of 11 patients with recurrent cancer or high-grade dysplasia had lesions locallized to the resection site [22]. In our study, size of adenomas greater than 1 cm did not increase the risk of advanced adenoma at surveillance colonoscopy, which is in line with Saini’s meta-analysis [32]. Incomplete removal or overlooked adenoma [21, 22] may account for these contradictory observations.

Adenoma number ≥ 3 has been shown to increase the risk of recurrent advanced adenoma. Relative risk for this parameter is between 1.5 and 5.0 [22, 32, 34–36], using a single adenoma as reference. Our findings agree with previous studies in terms of an odds ratios of 2.45 (1.22–4.93) upon univariate analysis and 2.11 (1.00–4.43) on multivariate analysis, respectively, when comparing adenoma number ≥ 3 to adenoma number ≤ 2. Male gender is also associated with advanced adenoma recurrence in his study, with an odds ratio of 3.09. This is consistent with Zhang’s study [17].

The natural history of colonic adenoma is still elusive. Two longitudinal follow-up studies on small polyps (6–9 mm) using computed tomography found a tumor progress rates of 22% [42] and 35% [43] during follow-up periods of 8 and 3 years, respectively. Advanced disease was seen in 47% of progressive polyps [43], which is similar to the rate of 40% in our patient group with baseline adenoma number ≥ 3. The high incidence rate of recurrent and advanced adenoma in patients with multiple lesions is hard to explain solely based on incomplete resection. Multiple small polyps not detected at baseline colonoscopy might progress slowly to become detectable at surveillance colonoscopy. Multiplicity or polyclonicity in patients with adenoma number ≥ 3 is a reasonable explanation, but a longer observation period with other non-invasive study modalities such as computed tomography or capsule endoscopy to detect missed adenomas may be required to elucidate the natural history of recurrent adenomas. The surveillance period recommended by the United States Preventive Services Task Force (USPSTF) is 3 years after removal of advanced adenoma, traditional serrated adenoma, or advanced sessile serrate adenoma [44]. The European guidelines [45] recommend a more aggressive surveillance at 1 year for high-risk polyps (≥ 20 mm). For treatment with piecemeal resection, Walsh et al. found a rate of 14% polyp recurrence after at least one negative examination, and the rate of CRC development was 17% among 65 patients with large, flat polyps [46]. A second look examination for patients who undergo piecemeal resection or suspected incomplete resection may be warranted.

There were a few limitations associated with our study. We did not take into account the impact of diet or lifestyle change such as abstinence from smoking, alcohol, or reduction of body mass index after polypectomy. These changes may influence the recurrence of adenoma or advanced adenoma [47, 48]. We also did not include low-risk patients as a control group, and the surveillance time of patients recruited was not uniform. These factors may influence the rate of adenoma recurrence.

## Conclusion

Colon polyp recurrence is common among patients who exhibit high grade dysplasia at index colonoscopy. Number of polyps ≥ 3 and gender (male) are traits that carry a higher risk for future occurrence for both typical and advanced adenoma.

## Abbreviations

HGD: High grade dysplasia
CRC: Colorectal cancer

## Availability of data and material

Colonoscopy and pathology reports from September 1998 to August 2007 were obtained from the electronic medical records of Chang Gung Memorial hospital (Keelung, Taiwan). The datasets generated and/or analyzed during this study are not publicly available given our commitment to patient privacy rights. However, anonymous data may be requested from the corresponding author for valid use.
